# Establishing age- and sex-specific anthropometric growth references standards for South Punjab adolescents utilizing the LMS method: findings from the Pakistani population

**DOI:** 10.3389/fpubh.2024.1417284

**Published:** 2024-09-12

**Authors:** Liu Long, Syed Danish Hamdani, Syed Muhammad Zeeshan Haider Hamdani, Jie Zhuang, Haris Khurram, Syed Ghufran Hadier

**Affiliations:** ^1^School of Physical Education, Suzhou University, Suzhou, Anhui, China; ^2^Division of Olympic Sports, China Swimming College, Beijing Sport University, Beijing, China; ^3^School Education Department, Government of Punjab, Multan, Pakistan; ^4^School of Exercise and Health, Shanghai University of Sport, Shanghai, China; ^5^Department of Sports Sciences, Bahauddin Zakariya University, Multan, Pakistan; ^6^Department of Mathematics and Computer Science, Faculty of Science and Technology, Prince of Songkla University, Pattani Campus, Pattani, Thailand; ^7^Department of Sciences and Humanities, National University of Computer and Emerging Sciences, Faisalabad Campus, Chiniot, Pakistan; ^8^School of Physical Education, Shanxi University, Taiyuan, Shanxi, China

**Keywords:** anthropometric growth, normative references standards, health evaluation, growth and development, LMS method, growth charts, Pakistani adolescents

## Abstract

**Background:**

The physical health of adolescents is crucial for the prosperity and sustainable development of a nation. Developing specific growth standards is essential for prioritizing the wellbeing of the youth of Pakistan. This study aimed to establish normative standards for height, weight, and body mass index (BMI) among 12- to 16-year-olds in South Punjab, facilitating accurate health assessments and tailored interventions.

**Method:**

This study utilized a cross-sectional design and stratified random sampling to select 2,970 adolescents (49.73% boys and 50.26% girls) aged 12–16 years from South Punjab, Pakistan. Anthropometric measurements, including height, weight, and BMI, were collected. The data were stratified by age and sex, and smoothed percentile curves were computed using the LMS method, which incorporates the L (γ-lambda), M (μ-mu), and S (δ-sigma) parameters. The results were compared to international references to provide a comprehensive analysis.

**Results:**

The results highlight sex-specific trends in anthropometric indicators among adolescents. Boys exhibited higher mean values in height (160.50 ± 11.50 cm), weight (45.02 ± 9.78 kg), and BMI (17.30 ± 2.41) than girls (158.57 ± 9.34 cm, 41.00 ± 7.89 kg, and 16.29 ± 2.82, respectively). Growth patterns indicate boys grow faster in height and weight between ages 12 and 14, whereas girls show slower annual increases. Comparative analysis with international standards reveals that boys’ height and weight were generally lower than international medians (P50th), whereas girls’ height was comparable or higher. BMI values for both sexes were lower than international norms, reflecting unique regional growth patterns.

**Conclusion:**

This research establishes updated age- and sex-specific normative reference standards for adolescents in South Punjab, Pakistan. The study revealed that Pakistani adolescent boys exhibit higher mean values in height, weight, and BMI than girls, with faster growth rates between ages 12 and 14. Compared to international standards, Pakistani adolescents show lower BMI values, highlighting unique regional growth patterns. These standards have practical applications in screening, monitoring, and health strategy planning, contributing to efforts to promote a healthier future for the population. Future studies are recommended to utilize these local growth references for health surveillance and treatment in the local population.

## 1 Background

Adolescence is a crucial phase for achieving physical and cognitive potential, requiring adolescents to experience healthy growth and development (1). Ensuring healthy growth involves providing support across essential domains, including physical, cognitive, and emotional areas, which are critical from birth through to adulthood (2). A comprehensive approach significantly shapes the overall wellbeing of young individuals as adolescence is a key indicator of future health (3). Growth monitoring is an essential component of global health services for children and adolescents, involving regular assessments of height, weight, body mass index (BMI), and other anthropometric indicators (4). This practice helps healthcare providers identify deviations in growth patterns using established growth references for effective tracking. Additionally, growth monitoring serves as an indirect method for evaluating the current health and nutritional status of communities, aiding in formulating strategies for future health improvements (5). In pediatric health, the lack of a universally accepted standard for assessing the growth and health status of children and adolescents leads to inconsistencies. The three recognized standards for assessing the growth and health status of children and adolescents, the CDC, IOTF, and WHO differ in their categorization within weight categories, leading to variations in reported prevalence rates across studies (6).

In Pakistan, adolescent growth monitoring primarily uses anthropometric data from the WHO and CDC. However, variations in the Human Development Index (HDI) across developed, developing, and underdeveloped countries suggest that generalized growth charts may not be appropriate for all regions (7, 8). Studies have demonstrated significant differences between locally developed reference values and those of the CDC and WHO, indicating the need for population-specific growth curves (7, 9–12). Children’s and adolescents’ growth patterns change over time due to various ecological, environmental, and genetic factors, making it important for each country and region to use population-specific growth references and regularly update these standards (11, 13). In Pakistan, there is an urgent need to develop updated growth reference standards for children and adolescents due to rising childhood obesity and the lack of national epidemiologic data, particularly in South Punjab province (13–15). Currently, Pakistan relies on the outdated National Center for Health Statistics reference from 1977 for child growth monitoring and lacks a national growth reference standard for adolescents (16).

Despite the utility of WHO and CDC references for international comparisons, localized standards are needed to enhance the understanding of growth and nutritional abnormalities, particularly in Punjab, Pakistan (13). Existing studies on the growth of children and adolescents in Pakistan have relied on WHO and CDC references, yet no validated national or regional growth standards for Pakistani adolescents have been established (9–11). The generalizability of these studies is limited due to small sample sizes and lack of validation (9, 10, 17). Khadilkar notes that global growth references, such as WHO and CDC, may inaccurately diagnose growth patterns in developing countries because they are based on global averages (18). Therefore, developing and establishing local age- and sex-specific anthropometric growth reference standards for Pakistani adolescents through the LMS method is a vital step toward understanding regional growth patterns and improving the health of this population (13). These percentiles, specifically intended to serve the South Punjab population, are not meant to be universal standards due to regional variations in socioeconomic, cultural, genetic, dietary, and health status (19–21). Comparing these local standards with international ones, such as WHO charts, will provide better insights into regional growth trends.

This pioneering study aimed to develop and validate normative reference values and growth charts for schoolgoing adolescents aged 12–16 years in South Punjab Province, Pakistan, focusing on height, weight, and BMI. The objectives were to (1) establish the percentile distribution of adolescents by age and sex and (2) compare these percentiles with international reference studies. Understanding adolescent growth in this region provides significant insights into their living conditions and aids in developing national growth references for Pakistani adolescents. Reliable growth data for adolescents in South Punjab Province will facilitate early detection of growth abnormalities and enable timely interventions, ultimately promoting optimal growth and development.

## 2 Methods

### 2.1 Study design

This cross-sectional study was conducted in the South Punjab Province during the 2019 academic year, using a stratified random sampling technique. This study was conducted as part of the Young Teen’s Assessment Active Lifestyle Involvement—PAKistan Study (YAALI-PAK) (22), which investigates the lifestyle habits and health outcomes of Pakistani adolescents (23). South Punjab was chosen for its diverse demographic and socioeconomic characteristics. The province was divided into three strata: Multan, Bahawalpur, and D.G. Khan. Stratified random sampling with equal allocation ensured proportional representation (24). Out of 360 high schools, 20 were randomly selected from each stratum, totaling 60 schools (16.67% of the total). Within each selected school, 50 students were randomly chosen, ensuring equal opportunity and maintaining representativeness.

#### 2.1.1 Sample size

The sample size was determined using the commonly employed equation: $n=\frac{{\left(Z\right)}^{2}PQ}{e^{2}}\times D$ (15, 25). In this equation, P is the proportion of the target population estimated to have a particular characteristic (0.4 or 40%), and Q is 1-P, calculated as 0.6. The Z is 1.96, reflecting a 95% confidence level, e is the level of precision (0.05), and 𝐷 is the design effect, assumed to be 1.

Using the sample size calculator with the assumption that 40% of the target population is enrolled in high school, the initial calculation suggested a smaller sample size. However, to enhance the generalizability and reliability of the findings, the sample size was increased to 3,000 adolescents aged 12–16. Utilizing the equal allocation method this sample was equally divided between boys (50%) and girls (50%) to meet the research objectives (15, 22). After accounting for non-responsive or incomplete responses, the final sample included 2,970 participants aged 12–16 years. Targeting the 12–16 age range captures characteristics of both early adolescence and late adolescence, thus effectively representing the broader adolescent population despite the narrower age range. This approach provides a balanced view of adolescent development.

A larger sample size generally establishes normative standards more effectively and yields more accurate and reliable results. The inclusivity of the larger sample ensures that the findings accurately reflect the broader adolescent population. Additionally, the power of sample size exceeded 80%, ensuring a high level of precision at a 5% significance level (23, 26). A statistical power of 80% is commonly considered adequate for most studies, ensuring a high probability of identifying true effects.

Sample size calculators and formulas typically provide sample sizes for achieving a 5% level of significance and a 95% confidence level. In this study, the sample size was more than sufficient, as the recommended sample sizes for these criteria are lower than the actual sample size used. This means the design of the study meets and exceeds standard statistical criteria, reinforcing the reliability and validity of the findings. The sample size of the study of 3,000 respondents ensures reliable and statistically valid results, with high power and low risk of type I error, providing a high level of precision in the findings.

#### 2.1.2 Inclusion and exclusion criteria

Participants were selected based on stringent criteria to ensure the credibility and applicability of the findings to the intended population. Eligible individuals had no disabilities, severe abnormalities, or clinical pathologies. This eligibility was confirmed through schools’ medical records and physical examinations. Individuals with pre-existing medical conditions, physical or cognitive disabilities, or those who sustained recent injuries were excluded. These measures maintained the integrity of the study.

### 2.2 Procedures

Before commencing data collection, we obtained necessary approvals and consents from school principals and parents, adhering to ethical standards for research involving minors. To ensure privacy, we implemented strict measures, including a prohibition on photography and sex-specific restrictions for research assistants in schools. We assembled a team of 12 research assistants from the Department of Sports Sciences at Bahauddin Zakariya University, who underwent comprehensive training in a workshop. This training focused on measurement techniques and protocols to ensure accuracy and consistency in data collection, in accordance with a protocol developed by the National Youth Fitness Survey (NYFS) by the Centers for Disease Control and Prevention, as detailed in an earlier study (27).

Data collection was conducted during the 2019 academic year in schools across the South Punjab Region, Pakistan. The schedule was carefully planned to coincide with the schools’ academic calendars and minimize disruption. Preparations for each school visit included confirming class availability and communicating testing requirements 2 days prior to the visits. On the designated collection day, students were randomly selected for participation. The data collection involved measurements of height and weight and the calculation of BMI. Demographic details such as school, class, sex, and date of birth were also gathered from school records. The assessment of anthropometrics and BMI was completed efficiently within one working day.

#### 2.2.1 Measures

Demographic data, including age and sex, were collected through self-reports by students or obtained from school records. Participants were measured barefoot, with their heads and shoulders aligned perpendicular to the scale. To ensure accuracy, all participants removed any accessories and shoes before measurements.

##### 2.2.1.1 Height measurement

Height was measured in centimeters (cm) as a standard indicator of growth and development in adolescents. Combined with weight, it effectively evaluates the BMI status of students. The measurement was conducted using a height-weight scale (DT-150 Height And Weight Scales, Shanghai, China) with the metrical rod of this scale used to measure height. The subjects stood barefoot, back against the wall, on a flat surface, maintaining a straight posture with arms relaxed at their sides and heels together, toes slightly apart at a 60-degree angle. Measurements were recorded to the nearest 0.1 cm for precision.

##### 2.2.1.2 Weight measurement

Weight was measured in kilograms (kg). Combined with height, it effectively evaluates the students’ BMI status. Weight was measured using the same height–weight scale (DT-150 Height And Weight Scales, Shanghai, China). Before testing, the sensitivity of the scale was checked by adding an extra 100 g weight, confirming the sensitivity of the instrument with a corresponding increase of 0.1 kg on the display. Accuracy was verified using standard weights of 10 kg, 20 kg, and 30 kg, with inspection errors not exceeding 0.1 kg. During the test, male and female subjects stood barefoot on the scale, maintaining body balance. Readings were recorded to one decimal place in kilograms.

##### 2.2.1.3 Body mass index

BMI is calculated by dividing weight in kilograms by the square of height in meters. It is a commonly used international standard to measure obesity and health, primarily for statistical purposes (15). BMI provides a neutral and reliable indicator for comparing and analyzing the health effects of weight among individuals of different heights (28).

$$BMI=\mathrm{weight}\;\left(\mathrm{kg}\right)/\mathrm{height}\;{\left(m\right)}^{2}$$

### 2.3 Statistical analysis

In this study, we employed the LMS technique to develop age- and sex-specific height, weight, and BMI percentiles based on our data, using RStudio version 4.1.3. The LMS method, incorporating the parameters L (λ, skewness), M (μ, median), and S (σ, coefficient of variation), is widely employed for developing growth percentiles globally (29, 30). The generated references for height, weight, and BMI were cross-validated using a back-generation test. Descriptive statistical analysis was conducted using SPSS version 21.0, including the calculation of percentages, frequencies, mean values, and standard deviations. Statistical significance was determined at the 5% level (*p* < 0.05).

#### 2.3.1 Back generation test

To validate the normative reference standards developed using the LMS method, the study employed a back-generation test for cross-validation, ensuring its scientific credibility (22). This involved comparing the 50th percentile (P50) values from randomly extracted datasets, confirming the accuracy of the method across various age groups and sexes with mean absolute error rates within acceptable limits. Additionally, the study evaluated forecasting accuracy using the mean absolute percentage error (MAPE), a measure introduced by Lewis in 1982, to assess forecast precision. MAPE classifies forecasting accuracy as follows: below 10% as “Highly accurate,” 10–20% as “Good,” 20–50% as “Reasonable,” and above 50% as “Inaccurate” (31). This dual approach enhances methodological rigor, ensuring precise forecasting and robust establishment of normative standards.

#### 2.3.2 Ethical approval

Ethical approval for this study was obtained from the School of Exercise and Health at Shanghai University of Sport (Approval number: 1716516032) in September 2018 and by the Ethics Advisory Committee of Bahauddin Zakariya University, Multan (Reference: 374/UREC/2018). To ensure adherence to ethical standards and protect participants, written consent was obtained from parents. In cases where parental consent was unavailable, approval was secured from the school principals, who were responsible for the students. This process ensured compliance with both institutional guidelines and the principles outlined in the Declaration of Helsinki, thereby safeguarding participant rights and welfare and maintaining the highest ethical standards throughout the study.

## 3 Results

In this study, we analyzed a sample of 2,970 adolescents, aged 12–16 years, from South Punjab Province, Pakistan, comprising 49.7% boys and 50.3% girls. We stratified the sample by age and sex. The sample was equally distributed across all age groups (12–16 years), assigning 20% of the population to each age group (see Table 1).

**Table 1:** Demographic analysis as per age and sex.

Age groups (years)	Boys	Girls	Total
12 N (%)	291 (19.70)	299 (20.24)	590 (19.90)
13 N (%)	295 (19.97)	298 (20.17)	593 (20.00)
14 N (%)	298 (20.17)	296 (20.04)	594 (20.00)
15 N (%)	298 (20.17)	300 (20.31)	598 (20.10)
16 N (%)	295 (19.70)	300 (20.31)	595 (20.00)
Total N (%)	1,477 (100)	1,493 (100)	2,970 (100)

The results of the comparison of sex-specific anthropometric indicators are presented in Table 2. The mean height of the male participants was 160.50 ± 11.50 cm, while that of the female participants was 158.57 ± 9.34 cm. The mean weight of the male participants was 45.02 ± 9.78 kg, and the mean weight of the female participants was 41.00 ± 7.89 kg. The mean BMI of the male participants was 17.30 ± 2.41, while the mean BMI of the female participants was 16.29 ± 2.82. The table demonstrates that, on average, male participants had higher anthropometric characteristics than female participants.

**Table 2:** Sex-specific anthropometric characteristics.

Component	Total (*n* = 2,970)	Boys (*n* = 1,477)	Girls (*n* = 1,493)	*p*-value
Height (cm)	159.53 ± 10.51	160.50 ± 11.50	158.57 ± 9.34	<0.001
Weight (kg)	43.00 ± 9.10	45.02 ± 9.78	41.00 ± 7.89	<0.001
BMI (kg/m^2^)	16.80 ± 2.67	17.30 ± 2.41	16.29 ± 2.82	<0.001

The data are presented as mean and standard deviation. BMI (kg/m^2^): Body mass index.

### 3.1 Back-substitution test

The back-substitution test was employed to evaluate the cross-validation of normative standards for anthropometric indicators. This study involved randomly extracting a small dataset to compare P50 values of the large (actual values) and small (fitted values) datasets, using the LMS method to establish the normative standard. By assessing the degree of coincidence between datasets generated through random sampling, we ensured the validity and applicability of the normative standards.

Table 3 presents the results of the back-substitution test for height, weight, and BMI, stratified by age and sex. The mean absolute percentage error difference between actual and fitted values was within an acceptable range for both boys and girls. For boys, the actual and fitted values for height were nearly identical across all ages (12–16 years), with MAPE values very close to zero, indicating an excellent fit. Similarly, for girls, the actual and fitted height values closely matched, with MAPE values of 0.00, indicating a perfect fit.

**Table 3:** Back-substitution test of height, weight, and BMI stratified by age and sex.

	Age	Male			Female		
		Actual value	Fitted value	MAPE	Actual value	Fitted value	MAPE
Height (cm)	12	149.72	150.00	0.00	152.16	152.00	0.00
	13	158.62	159.50	−0.01	157.87	159.00	−0.01
	14	164.74	165.00	0.00	161.68	162.00	0.00
	15	166.11	165.00	0.01	162.38	162.00	0.00
	16	167.79	168.00	0.00	162.65	162.00	0.00
	Average			−0.001			0.000
Weight (kg)	12	37.38	35.90	0.04	35.70	33.85	0.05
	13	41.79	41.50	0.01	37.73	36.00	0.05
	14	45.28	45.23	0.00	40.93	41.00	0.00
	15	47.58	47.00	0.01	44.02	43.00	0.02
	16	49.68	49.00	0.01	44.12	43.50	0.01
	Average			0.015			0.028
BMI	12	16.59	16.45	0.01	15.26	14.67	0.04
	13	16.81	16.54	0.02	15.71	15.82	−0.01
	14	17.03	17.19	−0.01	15.73	15.60	0.01
	15	17.26	16.70	0.03	16.74	16.36	0.02
	16	17.48	17.15	0.02	16.81	16.52	0.02
	Average			0.014			0.016

MAPE, mean absolute percentage error.

For boys, actual and fitted weight values also showed a high degree of accuracy, with MAPE values ranging from 0.00 to 0.01, suggesting minimal error. Girls exhibited slightly higher MAPE values for weight, ranging from 0.00 to 0.04, with an average MAPE of 0.028, indicating a small but slightly higher prediction error than boys. Boys’ actual and fitted BMI values were very close, with MAPE values ranging from 0.00 to 0.04 and an overall average MAPE of 0.014, reflecting very low prediction errors. Similarly, the BMI values of girls closely matched the actual and fitted values, with MAPE values ranging from 0.00 to 0.03 and an average MAPE of 0.016, indicating low prediction errors comparable to boys.

The results indicate that the fitted models for height, weight, and BMI were highly accurate for both boys and girls across ages 12 to 16 years. The low MAPE values across all categories suggest that the predictive models used for back-substitution were reliable and precise, with height predictions showing almost no error and slightly higher but still minimal errors for weight and BMI predictions. The overall average MAPE values were 0.014 for boys and 0.016 for girls, indicating excellent predictive performance of the models. The back-substitution approach ensured that the established normative standard was based on sound scientific principles.

### 3.2 Normative reference standard

**Figure 1:**
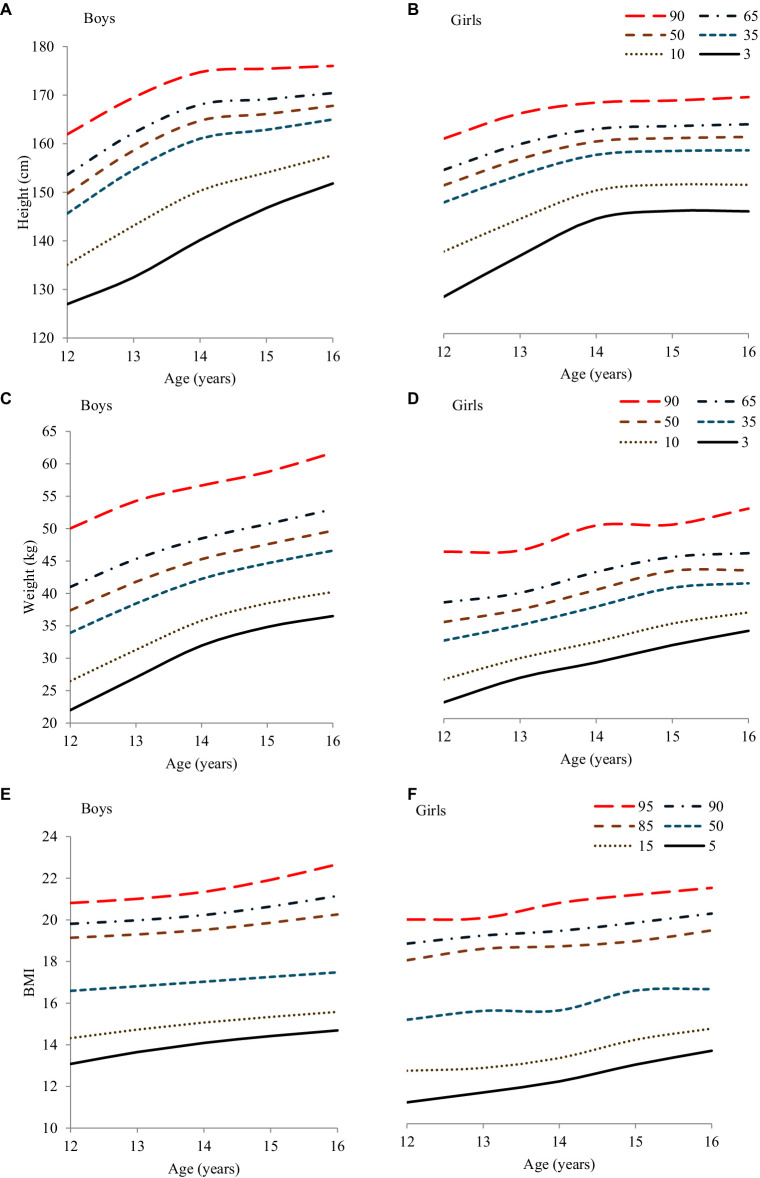
Height, weight, and BMI **(A,C,E)** boys, **(B,D,F)** girls smoothed percentile curves for South Punjab.

Percentile values and height curves by age and sex for boys and girls, calculated using the LMS technique, are presented in Table 4 and Figures 1A,B. Across the sampled age range, the median (P50th) height values for boys were between 149.72 cm and 167.79 cm, whereas for girls, they ranged from 152 cm to 166.5 cm. Between the ages of 12 and 16 years, the median growth in height for boys was 18.07 cm, compared to 10.49 cm for girls. Additionally, height ranges and annual growth trends for boys and girls were analyzed. Boys exhibited an average annual height growth of 6–9 cm between the ages of 12 and 14 years. In contrast, girls showed an average annual height increase of 4.5–5 cm during the same period. From ages 14 to 16 years, both boys and girls grew approximately 1–2 cm per year. Overall, boys’ height percentiles were generally higher than girls’, except at the age of 12 years, where girls’ percentiles were higher.

**Table 4:** Height (cm), weight (kg) percentile by age and sex in adolescents aged 12–16 years.

	Percentile	L	S	M	P3	P10	P35	P50	P65	P90
Height (cm)	**Boys**									
	12	3.020	0.069	149.72	126.98	135.08	145.63	149.72	153.60	161.93
	13	5.231	0.062	158.62	132.49	143.13	154.63	158.62	162.23	169.52
	14	6.000	0.055	164.74	140.16	150.31	161.05	164.74	168.06	174.72
	15	5.021	0.049	166.11	146.77	154.04	162.85	166.11	169.13	175.43
	16	4.931	0.042	167.79	151.81	157.61	164.99	167.79	170.42	176.00
	**Girls**									
	12	5.344	0.060	152.16	128.00	137.81	148.45	152.16	155.51	162.29
	13	4.770	0.055	157.87	136.89	144.87	154.39	157.87	161.09	167.76
	14	4.924	0.045	161.68	144.91	151.07	158.78	161.68	164.39	170.10
	15	4.759	0.043	162.38	146.62	152.32	159.60	162.38	164.99	170.53
	16	4.179	0.045	162.65	146.52	152.26	159.75	162.65	165.39	171.28
Weight (kg)	**Boys**									
	12	0.531	0.246	37.38	21.98	26.47	33.92	37.38	41.00	50.03
	13	0.368	0.214	41.79	27.03	31.29	38.44	41.79	45.32	54.27
	14	0.205	0.179	45.28	31.94	35.80	42.24	45.28	48.49	56.66
	15	0.042	0.165	47.58	34.81	38.47	44.65	47.58	50.70	58.73
	16	−0.122	0.167	49.68	36.50	40.22	46.60	49.68	52.99	61.72
	**Girls**									
	12	0.301	0.226	35.70	22.65	26.36	32.69	35.70	38.90	47.13
	13	0.158	0.180	37.73	26.64	29.83	35.19	37.73	40.42	47.33
	14	0.062	0.179	40.93	29.13	32.48	38.20	40.93	43.84	51.40
	15	0.202	0.165	44.02	31.95	35.46	41.29	44.02	46.29	51.53
	16	0.515	0.126	44.12	34.27	37.27	42.00	44.12	46.89	54.15

Ages shown represent age at last birthday (e.g., Age 12 = 12.0–12.9, 13 = 13.0–13.9).

Regarding weight, Table 4 and Figures 1C,D display the centile values and curves for both sexes. Boys’ median (P50th) weight values ranged from 37.38 kg to 49.68 kg, whereas girls ranged from 35.70 kg to 44.12 kg. The total median weight gain between ages 12 and 16 was 12.3 kg for boys and 8.42 kg for girls. The annual weight gain trends showed that boys and girls experienced a yearly increase of approximately 1–4 kg and 2–3 kg, respectively, between the ages of 12 and 14 years. From 14 to 16 years, both boys and girls had a similar annual increase of approximately 1–2 kg. Overall, boys’ weight percentiles were higher than girls’ centiles.

The percentile values and curve for BMI for both sexes are shown in Table 5 and Figures 1E,F. The analysis reveals that both boys and girls experienced a steady increase in BMI over these years, with notable differences between the sexes.

**Table 5:** BMI percentile by age and sex in adolescents aged 12–16 years from South Punjab.

	Age	L	S	M	P5	P15	P50	P85	P90	P95
BMI	**Boys**									
	12	0.203	0.141	16.59	13.08	14.32	16.59	19.14	19.81	20.81
	13	−0.318	0.131	16.81	13.65	14.73	16.81	19.30	19.98	21.01
	14	−0.838	0.125	17.03	14.09	15.07	17.03	19.52	20.23	21.34
	15	−1.358	0.124	17.26	14.42	15.34	17.26	19.86	20.64	21.92
	16	−1.879	0.125	17.48	14.69	15.58	17.48	20.26	21.15	22.67
	**Girls**									
	12	0.283	0.202	15.26	11.08	12.68	15.26	18.27	19.11	20.33
	13	−0.173	0.172	15.71	11.58	12.82	15.71	18.85	19.52	20.40
	14	−0.495	0.168	15.73	12.14	13.32	15.73	18.97	19.75	21.17
	15	−0.234	0.159	16.74	12.99	14.25	16.74	19.23	20.17	21.58
	16	0.382	0.120	16.81	13.69	14.81	16.81	19.78	20.63	21.93

For boys, the median BMI (P50th) ranges from 16.59 at the age of 12 years to 17.48 at the age of 16 years, indicating an overall increase of 0.89 units. Similarly, girls show an increase in median BMI from 15.71 to 16.81 over the same age span, with a total rise of 1.1 units. This consistent upward trend in BMI is reflected in an approximate annual increase of 1 unit for both sexes.

Examining the BMI percentiles, boys generally have higher values than girls. For example, at the age of 12 years, the 5th percentile for boys is 14.06 and the 95th percentile is 21.34, whereas for girls, the corresponding values are 13.35 and 20.33. This pattern continues across all ages, with boys consistently exhibiting higher BMI percentiles. At age 16, boys’ BMI percentiles range from 15.37 (5th percentile) to 23.80 (95th percentile), whereas for girls, they range from 14.51 to 23.21.

Overall, the data indicate that as children in this South Punjab cohort grow from ages 12 to 16 years, both boys and girls experience a steady increase in BMI. However, male participants consistently display higher BMI percentile values than female participants, suggesting sex-based differences in body mass development during adolescence. This analysis underscores the importance of monitoring BMI trends to understand growth patterns and potential health implications in this population.

**Figure 2:**
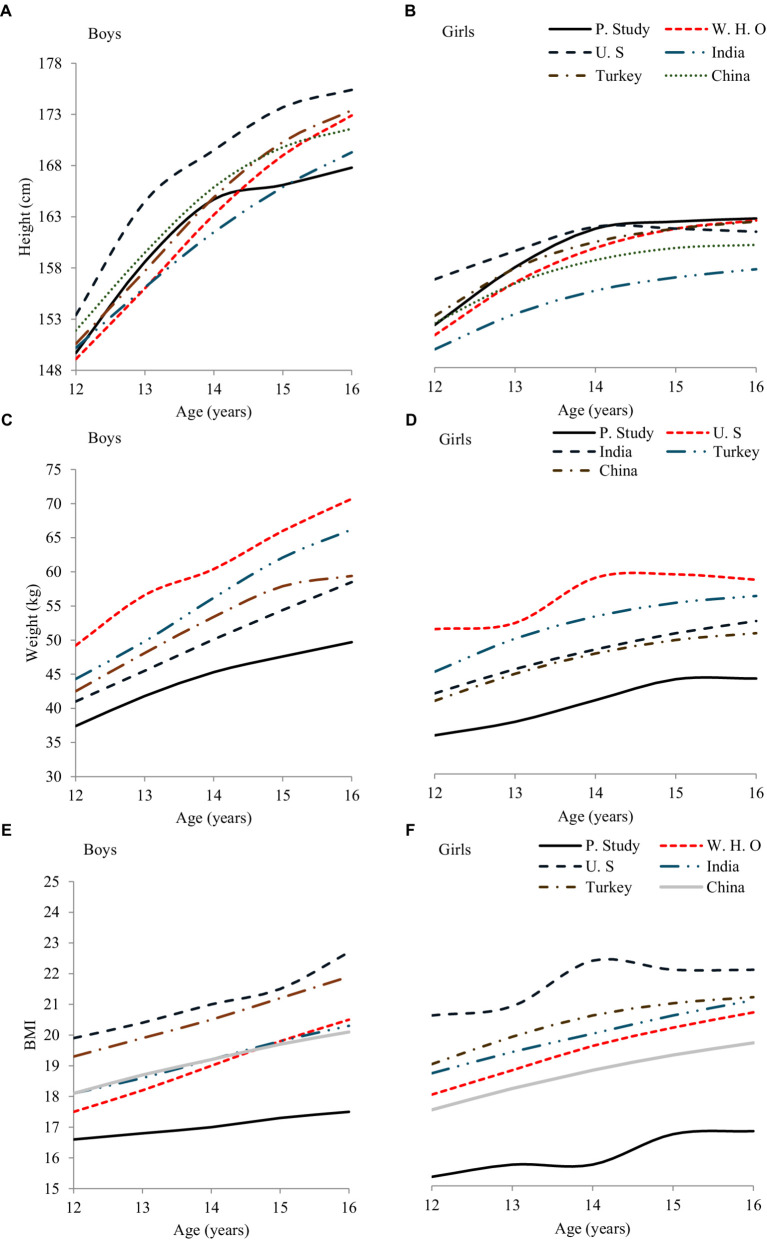
Height, weight, and BMI **(A,C,E)** boys, **(B,D,F)** girls comparison of P50 between present studies and published studies.

Table 6 and Figures 2A,B show the (P50^th^) centile values of height for both sexes in comparison with other research evidence from the WHO (32), US-CDC (33), India (34), Turkish (35), and China (36). The height centiles of the current study for 12–14-year-old boys were greater than WHO and India, while the rest of the age group is lower, similar to others. The percentiles of the current study were similar to international research between 13 and 14 years, except for the US-CDC 2012 (33). For girls, our data showed that the height of P50^th^ centiles from 12 to 16 years was higher than all the studied studies except the US-CDC study, where 12- to 14-year-old adolescent girls were taller. Comparing our findings with international research for 13–16-year-olds, the least difference was 1 cm, and the greatest was 8 cm.

**Table 6:** Height, weight, and BMI comparison of P50th between present studies and other published studies.

	Age (years)	Present study	WHO (2006)	US-CDC (2012)	India (2011)	Turkish (2015)	Chinese (2013)
Height (cm)	**Boys**						
	12	149.7	149.1	153.4	150.2	150.6	151.9
	13	158.6	156.0	164.5	156.1	157.7	159.5
	14	164.7	163.2	169.5	161.5	164.9	165.9
	15	166.1	169.0	173.7	165.9	170.3	169.8
	16	167.8	172.9	175.4	169.3	173.4	171.6
	**Girls**						
	12	152.2	151.2	156.7	149.8	153.1	152.4
	13	157.9	156.4	159.5	153.3	157.8	156.3
	14	161.7	159.8	161.9	155.6	160.4	158.6
	15	162.4	161.7	161.7	156.9	161.7	159.8
	16	162.7	162.5	161.4	157.7	162.4	160.1
Weight (kg)	**Boys**						
	12	37.4	–	49.2	41.0	44.3	42.5
	13	41.8	–	56.6	45.5	49.8	48.1
	14	45.3	–	60.4	50.1	56.2	53.4
	15	47.6	–	66.0	54.4	62.1	57.9
	16	49.7	–	70.7	58.5	66.2	59.4
	**Girls**						
	12	35.7	–	51.4	41.9	45.1	40.8
	13	37.7	–	52.3	45.5	50.0	44.8
	14	40.9	–	59.0	48.4	53.3	47.8
	15	44.0	–	59.5	50.8	55.3	49.8
	16	44.1	–	58.7	52.6	56.3	50.8
BMI	**Boys**						
	12	16.6	17.5	19.9	18.1	19.3	18.1
	13	16.8	18.2	20.4	18.6	19.9	18.7
	14	17.0	19.0	21.0	19.2	20.5	19.2
	15	17.3	19.8	21.5	19.8	21.2	19.7
	16	17.5	20.5	22.7	20.3	21.9	20.1
	**Girls**						
	12	15.3	18.0	20.6	18.7	19.0	17.5
	13	15.7	18.8	20.9	19.4	19.9	18.2
	14	15.7	19.6	22.4	20.0	20.6	18.8
	15	16.7	20.2	22.1	20.6	21.0	19.3
	16	16.8	20.7	22.1	21.1	21.2	19.7

Table 6 and Figures 2C,D show the (P50th) weight comparison for both sexes with other research evidence US-CDC (33), India (34), Turkish (35), and China (36). The median (P 50th) weight centiles for boys and girls aged 12 to 16 years in this study are lower than the United States, India, Turkey, and China. This comparison also reveals that our results differ significantly from those of all the studies included in this comparison.

Table 6 and Figures 2E,F illustrate the 50th percentile BMI comparisons for both sexes with international research data. Boys and girls aged 12 to 16 years in this study exhibit the lowest BMI values among all the compared studies from WHO (32), US-CDC (33), India (34), Turkish (35), and Chinese (36). The BMI ranges for Pakistani adolescents show notable differences when contrasted with those from other countries, highlighting distinct growth patterns and potentially different nutritional statuses.

The data from Table 6 and Figures 2A–F highlight significant differences in height, weight, and BMI among adolescents aged 12–16 years in the current study compared to those in international studies. While the height values for boys aged 12–14 years are higher than in some studies, they are generally lower for older ages. Height centiles of girls are consistently higher, except when compared to the US-CDC study. Both weights and BMI values of boys and girls are significantly lower than those reported by the US-CDC, India, Turkish, and Chinese studies. These findings underscore the importance of region-specific growth standards to accurately reflect the physical development and nutritional status of adolescents.

## 4 Discussion

Adolescence is a critical developmental phase marked by significant physical changes, and monitoring these changes is essential for predicting future health outcomes. Anthropometric assessments are prioritized in pediatric research to enhance understanding and prediction of adolescents’ health (37, 38). Literature highlights that the South Punjabi adolescent population lacked specific growth and normative standards (12, 39). Addressing this gap, this pioneering study employed the LMS method to establish localized normative standards and growth curves, which we subsequently compared with the globally recognized 50th percentile norms.

The LMS method is pivotal in this research, providing a robust statistical approach to construct smoothed percentile curves by estimating the L (skewness), M (median), and S (coefficient of variation) parameters (11). This method is widely regarded as superior for creating accurate growth charts due to its flexibility in modeling data that adhere to normal distribution after a Box–Cox power transformation (40). Literature supports the use of the LMS method for developing growth charts due to its ability to accommodate the age-specific skewness of anthropometric data, thus offering a more precise reflection of biological growth patterns (41).

This study aligns with previous findings, yet it notably highlights significant disparities in body weight, height, and BMI among different age and sex groups within the examined population (34, 35). This suggests that international references may not be universally applicable and necessitates the adoption of region-specific growth standards (42). Such discrepancies necessitate the adoption of region-specific growth standards to better address local public health needs that could help normalize the prevalence of underweight, overweight, and obesity.

### 4.1 Height

Height data from our study demonstrate a consistent growth pattern in both sexes until age 16, with notable deviations in growth trajectories compared to international norms from the WHO and CDC, among others (7, 43, 44). In particular, the growth trajectories for boys were more pronounced than girls during this period, which is consistent with existing literature (43, 44), including studies by WHO (32) and from regions like the US-CDC (33), India (34), Turkish (35), and China (36). However, a significant discrepancy was observed as girls from South Punjab at the age of 15 years recorded higher height measurements than these international reference standards from WHO (32), US-CDC (33), India (34), Turkish (35), and China (36). This is a significant discrepancy as it suggests that regional genetic, nutritional, or environmental factors may influence growth patterns differently in South Punjab compared to other regions.

The average heights recorded were 160.50 ± 11.50 cm for boys and 158.57 ± 9.34 cm for girls. The annual growth rates varied, with a sharper increase observed between ages 12 and 14 years and a more gradual growth from 14 to 16 years. Our results further revealed that boys generally exhibited higher growth centiles, except at the age of 12 years where girls surpassed boys. Height growth trends, as illustrated in our growth curves, showed consistent increases with age. However, when compared to the 50th percentile values from the USA-CDC (33), WHO (32), Chinese (36), and Turkish (35) studies, adolescents from South Punjab were found to be consistently shorter, ranging from 1 to 8 cm below than above-mentioned studies standards. This discrepancy highlights the importance of developing region-specific growth standards. The implications are significant for public health policies, as adopting international standards may not accurately reflect the growth patterns of South Punjab adolescents, potentially leading to misclassification of underweight, overweight, and obesity cases. Region-specific growth charts are crucial for accurate assessment and intervention.

### 4.2 Weight

Our study identified a significant increase in body weight from ages 12 to 16 years, aligning with findings from other international research, including studies from the US-CDC (33), India (34), Turkish (35), and China (36, 43). However, a critical discrepancy emerges as our study recorded the lowest average weight levels than these countries, with all groups peaking by age 16. This difference underscores potential variations in genetic, dietary, or lifestyle factors influencing adolescent growth patterns across different regions. The data indicate that both boys’ and girls’ weights progressively increase with age, and the annual weight increase observed was 1–4 kg for boys and 2–3 kg for girls aged 12–14 years, and 1–2 kg for both sexes from 14 to 16 years, these increments are consistent with existing literature (7, 43–45). However, our findings also suggest a lower rate of increase than the cohorts in the aforementioned countries, particularly during the critical growth phase of 14 to 16 years. This slower growth rate may reflect underlying socioeconomic, nutritional, or health-related issues that could be unique to the population studied.

Additionally, our study’s centile values, especially for boys, were consistently higher than those for girls, aligning with general physiological trends observed globally. However, the growth trajectories illustrated in Figures 1C,D for both sexes show that the increasing trend in weight with age is less pronounced in our study population compared to international peers from USA-CDC (33); India (34), Turkish (35), and Chinese (36). This could indicate a need for targeted health interventions to address potential deficiencies or public health strategies to better align adolescent growth with global standards. Our comparison in Table 6 and Figures 2C,D further clarifies that while our centile values deviate from those reported in other studies (Table 6), except for the US-CDC (33), these variations call for a deeper examination into the factors contributing to lower weight outcomes in our demographic. Understanding these discrepancies is crucial for implementing effective public health policies and nutritional programs tailored to specific community needs.

### 4.3 BMI trends and comparisons

The P50th percentile BMI values in our study were different and lower than the referenced studies, indicating potential regional differences in growth patterns. Specifically, both boys and girls in our cohort exhibited significantly lower BMI values at ages 15 and 16 years, with maxima of 18.06 kg/m^2^ and 17.06 kg/m^2^ respectively, than similar age groups in studies from the WHO (46), US-CDC (33), India (34), Turkey (35), and China (36). For instance, the average BMI for boys and girls in our study was 17.30 ± 2.41 kg/m^2^ and 16.29 ± 2.82 kg/m^2^, respectively (Table 2), which are considerably below the 50th percentile values reported in these international studies. This suggests a regional variation in adolescent BMI, potentially influenced by local dietary, genetic, and environmental factors. Boys and girls aged 14–16 years showed an annual BMI increase of approximately 1 kg/m^2^. Generally, Table 5 shows higher centile values for boys than girls. Figures 1E,F illustrates BMI curves indicating an increasing trend with age. The 12-16-year-olds in our study had the lowest BMI values among all the international studies, including WHO (32), US-CDC (33), India (34), Turkish (35), and Chinese (36).

Our results were comparable to most studies in Table 6, except for the US-CDC (33). Comparing P50th values of adolescents’ BMI with WHO, CDC, and others revealed lower P50th values in our study (Table 6, Figures 2E,F). This finding implies that the standard growth charts used globally may not be fully applicable to the adolescent population of South Punjab. Other epidemiological studies in Saudi Arabia, Turkey, Italy, and India also reported lower norm values than Iran (47), Italy (48), Turkish (35, 49), and India (50). This suggests a need for region-specific growth references. These discrepancies suggest that regional dietary habits, socioeconomic factors, and genetic differences might influence BMI variations. Lower BMI values in our sample might reflect nutritional deficiencies or different lifestyle patterns compared to the other countries studied. Further investigation is needed to understand these differences and to develop targeted nutritional and health interventions.

The observed variations in the 50th percentile values for height, weight, and BMI across sexes are consistent with findings from other global studies including the WHO (32), US-CDC (33), India (34), Turkish (35), and China (36). These variations may be attributed to the geographical representativeness of samples in each study. The WHO study by de Onis et al. (32) also faced issues with appropriate sample sizes (7, 32). Consistent with earlier studies by the WHO (32), US-CDC (33), India (34), Turkey (35), and China (36), height, weight, and BMI significantly increased with age. In this research, the maximum BMI was observed at the age of 16 years across all studies. Despite similarities, the US (33) and Turkish (35) populations showed significantly higher BMI values for several age groups between 12 and 16 years.

Although a significant difference has been found in height, weight, and BMI reference values of South Punjab, Province adolescents with others WHO (32), US-CDC (33), India (34), Turkish (35), and China (36). There is a significant difference in height, weight, and BMI reference values of South Punjab Province adolescents compared to other regions. Applying growth standards from other nations can lead to inaccuracies when children’s growth falls outside the specified percentiles (51). These findings underscore the importance of localized growth standards to address anthropometric disparities and highlight the limitations of applying global references to diverse populations (52).

Furthermore, regional differences in access to sports and physical activities significantly influence adolescent growth patterns globally, as well as within different regions of the same country (53, 54). For instance, adolescents in areas with extensive sports infrastructure typically exhibit lower obesity rates. In contrast, South Punjab, despite substantial investments in sports facilities, shows marked growth disparities due to fewer facilities compared to other regions. This regional imbalance underscores the role of physical activity infrastructure in public health (55). Other factors such as sedentary lifestyles, socioeconomic status, educational attainment, and cultural variations also play a critical role in these patterns (7, 56–58). Addressing these multifactorial influences is essential for developing targeted interventions to manage growth disorders and promote optimal BMI (59).

### 4.4 Strengths and limitations of the study

The primary strength of this research lies in its large sample size of 2,970 adolescents from South Punjab, Pakistan, which enhances the generalizability and reliability of the findings. Stratified random sampling across three divisions ensures diverse population inclusion, improving the representativeness of the results. Additionally, the appropriate statistical methods employed to construct percentile curves for height, weight, and BMI using the L (γ-lambda), M (μ-mu), and S (δ-sigma) parameters provide robust normative reference values. These generated percentiles are crucial for identifying atypical growth patterns that may indicate health risks, necessitating immediate medical consultation. Another significant advantage is the establishment of localized normative standards using the back-substitution method, aligning growth monitoring in South Punjab with international studies from various demographic settings including the United States, the WHO, India, Turkey, and China.

However, the focus of the study on 12–16-year-old adolescents in South Punjab, while useful for setting specific local norms, limits its broader applicability across Pakistan. The cross-sectional design restricts the ability to infer causality or track developmental changes over time. Additionally, potential sampling biases, despite efforts to mitigate them, and the exclusion of socioeconomic and dietary data limit the depth of understanding concerning the interplay between nutrition, social conditions, and adolescent growth. A significant limitation of our study is the exclusion of younger age groups from our growth reference investigation. This exclusion limits the ability to provide a comprehensive developmental trajectory from birth through adolescence. Consequently, our findings may not fully capture the early growth patterns that could inform adolescent growth trends. Despite this limitation, our study provides valuable insights into the specific age range studied. Future research to address this gap by incorporating longitudinal data from birth through adolescence. Furthermore, while international comparisons provide valuable insights, regional and ethnic growth disparities necessitate cautious application of these standards to the local context.

### 4.5 Medical implication of the study

The study offers significant medical implications by providing critical tools for assessing the physical growth and development of Pakistani adolescents. Establishing age- and sex-specific normative standards enables health professionals to identify individuals at risk of chronic conditions associated with suboptimal growth. These standards facilitate early detection and intervention, potentially enhancing health outcomes and reducing disease burden. Additionally, they are instrumental in evaluating the effectiveness of public health programs aimed at promoting adolescent wellbeing.

### 4.6 What did this study add?

This research establishes the latest age- and sex-specific normative reference standards for adolescents in South Punjab, marking a significant advancement in public health and adolescent medicine. These standards are integral to effective screening, growth monitoring, and public health surveillance, significantly enhancing adolescent health management and preventive care strategies.

## 5 Conclusion

This study established growth curves and normative standards for height, weight, and BMI, revealing significant sex and age differences and highlighting the distinct growth patterns of male and female students. International references generally showed higher values for older boys’ height (15–16), weight, and BMI for both sexes, except for boys’ height at 12–14 and girls’ height, where the values in this study were comparable or higher. These findings provide essential benchmarks for school physical education and health assessments, aiding in the early identification of chronic disease risks. Consequently, this research can significantly impact public health initiatives and improve health outcomes for Pakistani adolescents.

## Data Availability

The raw data supporting the conclusions of this article will be made available by the authors, without undue reservation.

## References

[1] Brown KA Patel DR Darmawan D. Participation in sports in relation to adolescent growth and development. Transl Pediatr. (2017) 6:150–9. doi: 10.21037/tp.2017.04.03, PMID: 28795005 PMC5532200

[2] Dahl RE Allen NB Wilbrecht L Suleiman AB. Importance of investing in adolescence from a developmental science perspective. Nature. (2018) 554:441–50. doi: 10.1038/nature25770, PMID: 29469094

[3] Brooks SJ Parks SM Stamoulis C. Widespread positive direct and indirect effects of regular physical activity on the developing functional connectome in early adolescence. Cereb Cortex. (2021) 31:4840–52. doi: 10.1093/cercor/bhab126, PMID: 33987673

[4] Lemes V Gaya AR Sadarangani KP Aguilar-Farias N Rodriguez-Rodriguez F Martins CMDL. Physical fitness plays a crucial mediator role in relationships among personal, social, and lifestyle factors with Adolescents' cognitive performance in a structural equation model. The Cogni-action project. Front Pediatr. (2021) 9:656916. doi: 10.3389/fped.2021.656916, PMID: 34195161 PMC8236613

[5] Victora CG Adair L Fall C Hallal PC Martorell R Richter L. Maternal and child undernutrition: consequences for adult health and human capital. Lancet. (2008) 371:340–57. doi: 10.1016/S0140-6736(07)61692-4, PMID: 18206223 PMC2258311

[6] Asato MR Terwilliger R Woo J Luna B. White matter development in adolescence: a DTI study. Cereb Cortex. (2010) 20:2122–31. doi: 10.1093/cercor/bhp282 20051363 PMC2923214

[7] Gómez-Galán R Pastor-Cisneros R Carlos-Vivas J Mendoza-Muñoz M Adsuar JC García-Gordillo MÁ. Normative values of height, bodyweight and body mass index of 12–17 years population from Extremadura (Spain). Biology. (2021) 10:645. doi: 10.3390/biology10070645, PMID: 34356500 PMC8301187

[8] Syed Ghufran H Medad Ali S Liu Y Haider HSMZ Danish HS. Assessment of the BMI among 8–12-year-old school students stratified by socioeconomic status from Multan, Pakistan: a cross-sectional study. J Manag Pract Hum Soc Sci. (2023) 7:39–49. doi: 10.33152/jmphss-7.6.4

[9] Asif M Aslam M Qasim M Altaf S Ismail A Ali H. A dataset about anthropometric measurements of the Pakistani children and adolescents using a cross-sectional multi-ethnic anthropometric survey. Data Brief. (2021) 34:106642. doi: 10.1016/j.dib.2020.106642, PMID: 33365371 PMC7749368

[10] Qaisar R Karim A. BMI status relative to international and national growth references among Pakistani school-age girls. BMC Pediatr. (2021) 21:535. doi: 10.1186/s12887-021-03017-z, PMID: 34852819 PMC8638413

[11] Shehzad MA Khurram H Iqbal Z Parveen M Shabbir MN. Nutritional status and growth centiles using anthropometric measures of school-aged children and adolescents from Multan district. Arch Pediatr. (2022) 29:133–9. doi: 10.1016/j.arcped.2021.11.010, PMID: 34955308

[12] Asif M Aslam M Mazhar I Ali H Ismail T Matłosz P. Establishing height-for-age Z-score growth reference curves and stunting prevalence in children and adolescents in Pakistan. Int J Environ Res Public Health. (2022) 19:12630. doi: 10.3390/ijerph191912630 36231930 PMC9566739

[13] Mushtaq MU Gull S Mushtaq K Abdullah HM Khurshid U Shahid U. Height, weight and BMI percentiles and nutritional status relative to the international growth references among Pakistani school-aged children. BMC Pediatr. (2012) 12:31. doi: 10.1186/1471-2431-12-31, PMID: 22429910 PMC3337223

[14] Roberts SB Dallal GE. The new childhood growth charts. Nutr Rev. (2001) 59:31–6. doi: 10.1111/j.1753-4887.2001.tb06973.x 11310773

[15] Liu Y Hadier SG Liu L Hamdani SMZH Hamdani SD Danish SS. Assessment of the relationship between body weight status and physical literacy in 8 to 12 year old Pakistani school children: the PAK-IPPL cross-sectional study. Children. (2023) 10:363. doi: 10.3390/children10020363, PMID: 36832492 PMC9955071

[16] de Onis M Wijnhoven TMA Onyango AW. Worldwide practices in child growth monitoring. J Pediatr. (2004) 144:461–5. doi: 10.1016/j.jpeds.2003.12.034 15069393

[17] Rodriguez-Martinez A Zhou B Sophiea MK Bentham J. Height and body-mass index trajectories of school-aged children and adolescents from 1985 to 2019 in 200 countries and territories: a pooled analysis of 2181 population-based studies with 65 million participants. Lancet. (2020) 396:1511–24. doi: 10.1016/S0140-6736(20)31859-6, PMID: 33160572 PMC7658740

[18] Khadilkar V Khadilkar A. Growth charts: a diagnostic tool. Ind J Endocrinol Metab. (2011) 15:166–S171. doi: 10.4103/2230-8210.84854, PMID: 22029020 PMC3183514

[19] Goel V. Why Are Some People Healthy and Others Not? The Determinants of Health of Populations. CMAJ: Can. Med. Assoc. J. (1995) 152:2004–2005.

[20] Tyakht AV Kostryukova ES Popenko AS Belenikin MS Pavlenko AV Larin AK. Human gut microbiota community structures in urban and rural populations in Russia. Nat Commun. (2013) 4:2469. doi: 10.1038/ncomms3469, PMID: 24036685 PMC3778515

[21] Tavella RA Fernandes CLF Schimith LE Volcão LM dos Santos M da Silva Júnior FMR. Factors associated with genetic damage — an analysis integrating human populations from southern Brazil. Environ Sci Pollut Res. (2022) 29:74335–45. doi: 10.1007/s11356-022-21089-x 35635668

[22] Hamdani SMZH Zhuang J Hadier SG Khurram H Hamdani SDH Danish SS. Establishment of health related physical fitness evaluation system for school adolescents aged 12–16 in Pakistan: a cross-sectional study. Frontiers. Public Health. (2023) 11:11. doi: 10.3389/fpubh.2023.1212396, PMID: 37829094 PMC10564982

[23] Hamdani S Jie Z Hadier SG Tian W Hamdani SDH Danish SS. Relationship between moderate-to-vigorous physical activity with health-related physical fitness indicators among Pakistani school adolescents: Yaali-Pak study. Sci World J. (2022) 2022:6402028–10. doi: 10.1155/2022/6402028, PMID: 36118288 PMC9473884

[24] Hadier SG Liu Y Long L Hamdani SMZH Khurram H Hamdani SD. Assessment of physical literacy in 8- to 12-year-old Pakistani school children: reliability and cross-validation of the Canadian assessment of physical literacy-2 (CAPL-2) in South Punjab, Pakistan. BMC Public Health. (2024) 24:1726. doi: 10.1186/s12889-024-19185-3, PMID: 38943131 PMC11212239

[25] William GC. Sampling techniques. 3rd Edition. New York: John Willey & Sons Inc. (1977).

[26] Suresh K Chandrashekara S. Sample size estimation and power analysis for clinical research studies. J Hum Reprod Sci. (2012) 5:7–13. doi: 10.4103/0974-1208.97779, PMID: 22870008 PMC3409926

[27] Hamdani SMZH Zhuang J Tian W Hadier SG. Normative reference standard for handgrip strength among adolescent students in South Punjab Pakistan: a cross-sectional study. J Bus Soc Rev Emerg Econ. (2021) 7:997–1009. doi: 10.26710/jbsee.v7i4.2064

[28] CDC. How is BMI used? Retrieved from Centers for Disease Control and Prevention. (2020). Available at: https://www.cdc.gov/healthyweight/assessing/bmi/adult_bmi/index.html.

[29] Tomkinson GR Lang JJ Tremblay MS Dale M LeBlanc AG Belanger K. International normative 20 m shuttle run values from 1 142 026 children and youth representing 50 countries. Br J Sports Med. (2017) 51:1545–54. doi: 10.1136/bjsports-2016-095987, PMID: 27208067

[30] Zhang F Yin X Bi C Li Y Sun Y Zhang T. Normative reference values and international comparisons for the 20-metre shuttle run test: analysis of 69,960 test results among Chinese children and youth. J Sports Sci Med. (2020) 19:478–88. PMID: 32874100 PMC7429421

[31] Meade N. Industrial and business forecasting methods, Lewis, C.D., Borough Green, Sevenoaks, Kent: Butterworth, 1982. Price: £9.25. Pages: 144. J Forecasting. 2:194–6. doi: 10.1002/for.3980020210

[32] de Onis M. WHO child growth standards based on length/height, weight and age. Acta Paediatr. (2006) 95:76–85. doi: 10.1111/j.1651-2227.2006.tb02378.x 16817681

[33] Fryar CD Gu Q Ogden CL. Anthropometric reference data for children and adults: United States, 2007-2010. Vital Health Stat 11. (2012) 252:1–48. 25204692

[34] Kanwar SC Sabharwal A Bhadra K Narang A. Nationwide reference data for height, weight and body mass index of Indian schoolchildren. Nat Med J India. (2011) 24:269–77, PMID: . 22680077

[35] Neyzi O Bundak R Gökçay G Günöz H Furman A Darendeliler F. Reference values for weight, height, head circumference, and body mass index in Turkish children. J Clin Res Pediatr Endocrinol. (2015) 7:280–93. doi: 10.4274/jcrpe.2183, PMID: 26777039 PMC4805217

[36] Zong X-N Li H. Construction of a new growth references for China based on urban Chinese children: comparison with the WHO growth standards. PLoS One. (2013) 8:e59569. doi: 10.1371/journal.pone.0059569, PMID: 23527219 PMC3602372

[37] Mateo-Orcajada A Abenza-Cano L Cano-Martínez A Vaquero-Cristóbal R. The importance of healthy habits to compensate for differences between adolescent males and females in anthropometric, psychological and physical fitness variables. Children. (2022) 9:1926. doi: 10.3390/children9121926, PMID: 36553369 PMC9777149

[38] Ning Y Yang S Evans RK Stern M Sun S Francis GL. Changes in body anthropometry and composition in obese adolescents in a lifestyle intervention program. Eur J Nutr. (2014) 53:1093–102. doi: 10.1007/s00394-013-0612-9, PMID: 24212451

[39] Hadier SG Yinghai L Long L Hamdani SMZH Khurram H Hamdani SD. Urdu translation and cross-cultural adaptation of Canadian assessment of physical Literacy-2 (CAPL-2) questionnaires: a reliability analysis in Pakistani children. New Dir Child Adolesc Dev. (2024) 2024:1–15. doi: 10.1155/2024/9611010

[40] Golle K Muehlbauer T Wick D Granacher U. Physical fitness percentiles of German children aged 9–12 years: findings from a longitudinal study. PLoS One. (2015) 10:e0142393. doi: 10.1371/journal.pone.0142393, PMID: 26544848 PMC4636306

[41] Cole TJ. Using the LMS method to measure skewness in the NCHS and Dutch national height standards. Ann Hum Biol. (1989) 16:407–19. doi: 10.1080/03014468900000532, PMID: 2802520

[42] Clarke P O’Malley PM Johnston LD Schulenberg JE. Social disparities in BMI trajectories across adulthood by gender, race/ethnicity and lifetime socio-economic position: 1986–2004. Int J Epidemiol. (2008) 38:499–509. doi: 10.1093/ije/dyn214 18835869 PMC2663716

[43] Rebato E. Crecimiento: una visión desde la Antropología Física. Rev Esp Antrop Fís. (2010) 31:85–110.

[44] Carrascosa Lezcano A Fernández García JM Fernández Ramos C Ferrández Longás A López-Siguero JP Sánchez González E. Estudio transversal español de crecimiento 2008. Parte II: valores de talla, peso e índice de masa corporal desde el nacimiento a la talla adulta. An Pediatr. (2008) 68:552–69. doi: 10.1157/13123287, PMID: 18559194

[45] Cadenas-Sanchez C Intemann T Labayen I Peinado AB Vidal-Conti J Sanchis-Moysi J. Physical fitness reference standards for preschool children: the PREFIT project. J Sci Med Sport. (2019) 22:430–7. doi: 10.1016/j.jsams.2018.09.227, PMID: 30316738

[46] de Onis M. Assessment of differences in linear growth among populations in the WHO multicentre growth reference study. Acta Paediatr. (2006) 95:56–65. doi: 10.1111/j.1651-2227.2006.tb02376.x, PMID: 16817679

[47] Mahayar A Asefzadeh S. Comparison of weight, height and BMI of Iranian girl students with NCHS standard. NCD Malaysia. (2005) 4:11–5.

[48] Cacciari E Milani S Balsamo A Spada E Bona G Cavallo L. Italian cross-sectional growth charts for height, weight and BMI (2 to 20 yr). J Endocrinol Investig. (2006) 29:581–93. doi: 10.1007/BF03344156, PMID: 16957405

[49] El Mouzan MI Al Herbish AS Al Salloum AA Foster PJ Al Omar AA Qurachi MM. Comparison of the 2005 growth charts for Saudi children and adolescents to the 2000 CDC growth charts. Ann Saudi Med. (2008) 28:334–40. doi: 10.5144/0256-4947.2008.334, PMID: 18779639 PMC6074495

[50] Sharma A Sharma K Mathur KP. Growth pattern and prevalence of obesity in affluent schoolchildren of Delhi. Public Health Nutr. (2007) 10:485–91. doi: 10.1017/S1368980007223894, PMID: 17411469

[51] Gaylis JB Levy SS Hong MY. Relationships between body weight perception, body mass index, physical activity, and food choices in Southern California male and female adolescents. Int J Adolesc Youth. (2020) 25:264–75. doi: 10.1080/02673843.2019.1614465

[52] Lee Y-H Liu C-T Shelley M Chang Y-C. Regional and geographical disparities in body mass index (BMI) among Chinese older adults: the Chinese longitudinal healthy longevity survey. J Appl Gerontol. (2021) 40:1116–25. doi: 10.1177/0733464820930963, PMID: 32538233

[53] Andreff W Szymanski S. Handbook on the economics of sport. Cheltenham, UK: Edward Elgar Publishing (2006). Available at: http://site.ebrary.com/id/10328487

[54] Reis AC Vieira MC Sousa-Mast FRD. “Sport for development” in developing countries: the case of the Vilas Olímpicas do Rio de Janeiro. Sport Manag Rev. (2016) 19:107–19. doi: 10.1016/j.smr.2015.01.005

[55] Punjab Sports Department. Sports Policy Punjab. (2018). Available at: https://sportsboard.punjab.gov.pk/system/files/Sports%20Policy%2001.pdf.

[56] Drenowatz C Eisenmann JC Pfeiffer KA Welk G Heelan K Gentile D. Influence of socio-economic status on habitual physical activity and sedentary behavior in 8- to 11-year old children. BMC Public Health. (2010) 10:214. doi: 10.1186/1471-2458-10-214, PMID: 20423487 PMC2873582

[57] Vancampfort D Van Damme T Firth J Hallgren M Smith L Stubbs B. Correlates of leisure-time sedentary behavior among 181,793 adolescents aged 12-15 years from 66 low- and middle-income countries. PLoS One. (2019) 14:e0224339. doi: 10.1371/journal.pone.0224339, PMID: 31725744 PMC6855478

[58] Khan MS Khan A Ali A Akhtar N Rasool F Khan H. Prevalence of risk factors for coronary artery disease in southern Punjab, Pakistan. Trop J Pharm Res. (2016) 15:195–200. doi: 10.4314/tjpr.v15i1.27

[59] Fan X Cao Z-B. Physical activity among Chinese school-aged children: national prevalence estimates from the 2016 physical activity and fitness in China—the youth study. J Sport Health Sci. (2017) 6:388–94. doi: 10.1016/j.jshs.2017.09.006, PMID: 30356592 PMC6189233

